# Improvement of *Candida parapsilosis* by genome shuffling for the efficient production of arabitol from l-arabinose

**DOI:** 10.1007/s10068-018-0369-2

**Published:** 2018-04-04

**Authors:** Monika Kordowska-Wiater, Urszula Lisiecka, Krzysztof Kostro

**Affiliations:** 1 Department of Biotechnology, Microbiology and Human Nutrition, University of Life Sciences in Lublin, Skromna 8, 20-704 Lublin, Poland; 2 Department of Epizootiology and Clinic of Infectious Diseases, University of Life Sciences in Lublin, Głęboka 30, 20-950 Lublin, Poland

**Keywords:** *C. parapsilosis* mutant, Fusant, Genome shuffling, Protoplast fusion, Arabitol

## Abstract

Arabitol is used in the food industry as a low-calorie sweetener. It is produced by yeasts during the biotransformation process of l-arabinose. Genome shuffling was performed in *Candida parapsilosis* DSM 70125, an efficient producer of arabitol, to obtain fusants with improved arabitol production ability. Four mutants from the parental library were used for the first round of genome shuffling. The best fusants, GSI-1 and GSI-10A, were subjected to a second round of genome shuffling. Finally, two fusants, GSII-3 and GSII-16, produced concentrations of arabitol that were 50% higher than that of the wild-type strain during selection culture. Under the optimal conditions established for *C. parapsilosis*, the two fusants produced 11.83 and 11.75 g/L of arabitol and were approximately 15–16% more efficient than the wild-type strain. Flow cytometry analysis showed that the ploidy of the new strains did not change.

## Introduction

Genome shuffling is a strain improvement technology that combines classical mutagenesis with recombination. It uses recursive recombination between multiple parents of each generation to create mutant populations with enhanced genetic diversity and improved phenotypes [1–3]. Compared to other improvement techniques, genome shuffling has the advantage of exploiting the full genetic diversity of a population and offers the possibility of combining useful mutations from many different individuals [4]. This engineering technique does not require expensive equipment and can be applied easily in every laboratory. Importantly, shuffled strains are not considered to be genetically modified organisms and can thus be used in the food industry [2]. Each genome shuffling procedure consists of the following stages: parental library construction, recursive protoplast fusion, and desired phenotype selection. Each of these steps can be performed in a variety of ways, and it is up to the researcher to decide which method should be used; often, the researcher takes into account the available laboratory equipment, effectiveness, and other factors. Detailed information about the variations of the procedure for different microorganisms (bacteria, fungi, yeasts, and Streptomyces) can be found in published reviews [1, 2].

Yeasts can be easily subjected to gene shuffling, and procedures for different strains have been reported in the literature. The best known of these yeasts is *Saccharomyces cerevisiae*, which has been shuffled to obtain isolates with improved ethanol tolerance and productivity [5–7], enhanced stress tolerance [8], increased production of inhibitors present in lignocellulosic hydrolysates [9], increased fermentation performance in terms of yeast viability, beer flavour, and fermentation time [10], improved bioethanol production under very high gravity conditions [11], and increased spent sulfite liquor tolerance [12]. The xylose-fermenting yeast *Pichia* (*Scheffersomyces*) *stipitis* is also a species of interest for improving ethanol production from lignocellulosic materials by genome shuffling [13–15]. Zhang and Geng [16] used a modified method of genome shuffling to combine *S. cerevisiae* cells with the *P. stipitis* genome to obtain isolates with better ethanol productivity. Another xylose-fermenting yeast, *Pachysolen tannophilus*, was genome shuffled to improve its tolerance to inhibitors in hardwood spent sulfite liquor [17]. The ethanologenic yeast *Candida krusei* was modified with this method to obtain fusants with a higher acetic acid tolerance and an improved ability to produce ethanol from xylose [18]. *Candida versatilis* and *Hansenula anomala* were genome shuffled to improve their tolerance of salt stress and the formation of flavour in soy sauce [19, 20]. In a study by Zhang et al. [21], *P. anomala* was shuffled to improve sugar alcohol (D-arabitol and ribitol) production from glucose.

Arabitol, a five-carbon sugar alcohol, can be used as an alternative, natural, low-calorie sweetener in the food industry. This polyol does not lead to dental cavities, and it significantly reduces adipose tissue in the body and prevents the deposition of fat in the digestive tract; thus, arabitol has the potential for use in the human therapeutics industry. l-arabitol can be produced by the biotransformation of l-arabinose. Many yeasts have been screened and engineered for polyol production [22]. Among them, *C. parapsilosis* DSM 70125 is considered to be a good producer of arabitol [23] and is also known to be amenable to genetic improvement. In this study, two-round genome shuffling was used to obtain modified yeast cells able to efficiently produce arabitol from arabinose. To the best of the authors’ knowledge, this is the first report detailing genome shuffling in *C. parapsilosis*.

## Materials and methods

### Yeast strain

*Candida parapsilosis* DSM 70125 was obtained from the German Collection of Microorganisms and Cell Cultures and was maintained on YPG agar slants (BTL, Łódź, Poland) at 4 °C and subcultured at 3-month intervals. Experimentally obtained mutants of *C. parapsilosis* were used as the parental library for the genome shuffling procedure, and the fusants obtained were maintained as native strains.

### Media

The following four media types were used:

- Growth medium (YPG) (g/L): yeast extract (10.0); peptone (10.0); glucose (20.0); agar (20.0).
- Solid and liquid selective media for mutants (g/L): l-arabinose (20.0); (NH_4_)_2_SO_4_ (5.0); KH_2_PO_4_ (5.0); yeast extract (5.0); agar (20.0) (optional); pH 5.5.
- Regenerative-selective medium for fusants (g/L): yeast extract (10.0); peptone (20.0); l-arabinose (20.0); agar (20.0); 0.6 M KCl (44.75); 0.025 M CaCl_2_ (2.77); pH 6.0.
- Optimal medium (g/L): l-arabinose (32.5); (NH_4_)_2_SO_4_ (5.0); KH_2_PO_4_ (5.0); yeast extract (5.0); malt extract (5.0); pH 5.5.

### Mutagenesis and screening of mutants

A suspension of *C. parapsilosis* (1 × 10^7^ CFU/mL) was sonicated for 15 s (Vibra Cell, Sonics and Materials Inc., Newtown, CT, USA) to split the cells and buds and gently disintegrate the cell wall. Mutants of *C. parapsilosis* were obtained by exposing the sonicated suspension to UV irradiation (254 nm) (Philips TUV 30W/G30T8 lamp, Amsterdam, Holland) for 1 min at a vertical distance of 40 cm. Next, the cells were incubated in the dark for 2 h, diluted and seeded onto Petri dishes with selective agar. The dishes were incubated for 5 days, and colonies were isolated to select the most effective producers of arabitol. Cell viability was also calculated by comparing cell counts before and after mutagenesis. The mutants were inoculated into 10 mL of liquid selective medium in 50 mL Erlenmeyer flasks and incubated at 28 °C for 3 days on a rotary shaker (Infors HT Minitron, Infors AG, Bottmingen, Switzerland) at 150 rpm. Every 24 h, 1-mL samples were collected to measure the concentrations of l-arabinose and l-arabitol. Screening was performed in triplicate. The mutants that were the best producers of polyol were used as starting strains for the genome shuffling procedure.

### Genome shuffling

Suspensions of the five selected, freshly grown mutants with an optical density of 1° McFarland were prepared. Equal volumes of each isolate (0.2 mL) were mixed and collected by centrifugation at 11,200×*g* for 10 min. The mixture was suspended in 0.1 M phosphoric buffer with β-mercaptoethanol and incubated for 30 min at 28 °C. The cells were washed twice with phosphoric buffer. They were then suspended in this buffer with 0.6 M KCl and the enzyme lyticase (0.5%) (Sigma-Aldrich Co., St. Louis, MO, USA) and incubated with mild rotation for 60 min at 28 °C to obtain protoplasts. The formation of the protoplasts was monitored microscopically. Next, the protoplasts were washed twice (12 min, 7500×*g*) with phosphoric KCl buffer, and a control culture was prepared on regenerative-selective medium to determine the ability of the protoplasts to regenerate their cell wall. In the next step, the protoplast mixture was divided into two parts: one was inactivated thermally at 60 °C for 20 min, and the other was inactivated by exposure to UV light (254 nm) for 5.5 min. The conditions of inactivation were determined in an earlier experiment (data not shown). After inactivation, the control cultures were spread on regenerative-selective medium to check the effectiveness of the methods used. In the next step, the two suspensions of inactivated protoplasts were mixed, centrifuged (12 min, 5900×*g*), and suspended in 0.01 M Tris–HCl buffer with 40% polyethylene glycol (PEG 6000) and 0.01 M CaCl_2_ to be fused at 28 °C with mild rotation for 30 min. After being washed twice with a phosphoric buffer with KCl, the cells were diluted in the same buffer and spread onto Petri dishes containing regenerative-selective medium. They were then incubated at 28 °C for 5 days. All colonies were evaluated microscopically and inoculated onto YPG agar. These colonies were the products of the first round of genome shuffling. The procedure for the second round of shuffling of the fusants obtained in the first round was the same as above.

### Selection of fusants

The fusants were inoculated into 20 mL of liquid selective medium in 50 mL Erlenmeyer flasks and incubated at 28 °C for 3 days on a rotary shaker (Infors HT Minitron, Infors AG) at 150 rpm. Every 24 h, 1-mL samples were collected to determine which isolates were the most efficient producers of l-arabitol. The selection experiment was performed in triplicate.

### Arabitol production by the most efficient fusants under optimal conditions

The selected fusants (GSII-3, GSII-16, GSI-1 and GSI-10A) and the wild-type strain (control) were cultivated under the optimal conditions determined by the response surface method (RSM) in a previous study by Kordowska-Wiater et al. [24]. Yeast cultures were incubated for 3 days in 100 mL of optimal medium in 500 mL Erlenmeyer flasks at 28 °C on a rotary shaker (Infors HT Minitron, Infors AG) at 150 rpm. Every 24 h, samples were collected to measure the concentrations of l-arabinose, l-arabitol and biomass and the pH of the medium. The biotransformation experiment was performed in triplicate.

### Determination of the functional stability of the fusants

After serial passaging, the stability of the fusants was examined in batch cultures under the same conditions as those used in the selection experiment. After 48 h of incubation, the samples were collected to measure the concentrations of l-arabinose and l-arabitol in the cultures.

### Flow cytometry (FCM)

Cells from the wild-type strain *C. parapsilosis* and the selected fusants obtained in the first and second rounds of genome shuffling (GSI-1, GSI-10A, GSII-1, GSII-3, GSII-8, GSII-10, GSII-12, GSII-13, GSII-16, and GSII-35) were incubated in 10 mL of YPG medium at 28 °C to achieve the exponential growth phase. The cells were collected by centrifugation (5900×*g*, 10 min) and washed with PBS buffer. Next, the cells were fixed in 2 mL of 70% ethanol at 4 °C for 20 min; then, the volume was replenished with 5 mL of sterile water, and the samples were sonicated for 10 s. After centrifugation, the pellets were re-suspended in 2 mL of 50 mM Tris, pH 7.8, and centrifuged. Next, the pellets were re-suspended in TE buffer with 100 μL of RNase (10 mg/mL stock) and incubated at 30 °C for 30 min; then, the samples were centrifuged and re-suspended in TE buffer. The samples were diluted one hundred times, and each sample was then supplemented with propidium iodide (500 μg/mL in H_2_O stock), which binds DNA quantitatively. The samples were incubated at 4 °C for 30 min. Fluorescence intensity was measured using an Epics XL flow cytometer (Beckman Coulter, FL, USA). Cells were gated according to the region determined by FSC and SSC, and a total of 10,000 events were analysed for each sample.

### Analysis of arabinose and arabitol

Supernatants of the samples collected after a 15-min centrifugation at 7500×*g* were deproteinized with acetonitrile and analysed using an HPLC system (Gilson Inc., Middleton, WI, USA) equipped with a refractive index detector (Knauer GmbH, Berlin, Germany) and a Bio-Rad Aminex Carbohydrate HPX 87H column (Bio-Rad Laboratories Inc., Hercules, CA, USA) thermostatic at 42°C. Sulfuric acid (0.05 M) was used as the mobile phase at a flow rate of 0.5 mL/min. Chromatograms were integrated and analysed using Chromax 2007 software, version 1.0a (Pol-lab, Poland). The yield of arabitol was calculated as the grams of arabitol per grams of arabinose consumed.

### Analysis of biomass and pH

Biomass was measured as the optical density (OD) at 600 nm using a BioRad Smart Spec Plus spectrophotometer (Bio-Rad Laboratories Inc.). Then, the dry cell weight (DCW) was determined with a previously prepared calibration curve that correlated OD values with DCWs. The biomass yield was calculated as the grams of DCW per grams of arabinose consumed. The pH of the cultures was monitored during incubation using an electronic pH meter (Hanna Instruments, RI, USA).

## Results and discussion

*C. parapsilosis* DSM 70125, a yeast with high arabitol production efficiency, has been investigated by Kordowska-Wiater et al. [23]. In that study, it produced 10–14 g/L polyol from 20 g/L l-arabinose, depending on the cultivation conditions, with a yield of 0.51–0.78 g/g. These observations were confirmed during the statistical optimization of the biotransformation of l-arabinose to arabitol. It was determined that the optimum conditions for arabitol production (in which 14.3 g/L arabitol was obtained) included a temperature of 28 °C, a rotation speed of 150 rpm, and a substrate concentration of 32.5 g/L [24]. Due to its efficiency, strain DSM 70125 was selected for modification by genome shuffling.

### Parental library of mutants and selection of isolates for genome shuffling

**Fig. 1:**
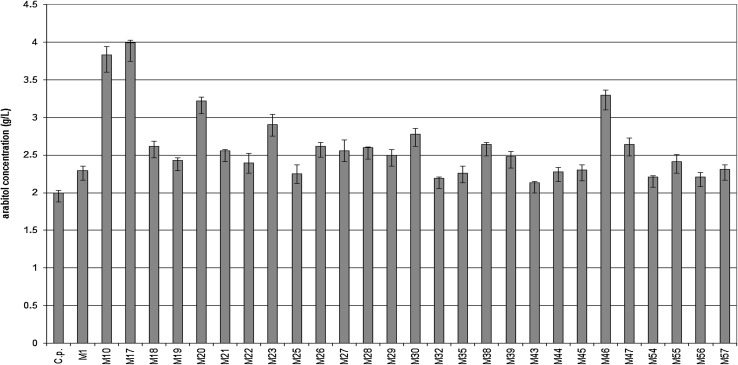
Comparison of arabitol production between *C. parapsilosis* DSM 70125 and its selected mutants. The mean values are from three experiments

Generally, a parental library is constructed using cell mutagenesis. UV irradiation is one of the preferred mutagens for yeasts. For examples, it was used by Shi et al. [5] and Wang and Hou [10] to obtain *S. cerevisiae* mutants and by Wei et al. [18] to produce *C. krusei* mutants. Zhang et al. [21] used UV irradiation together with the ARTP (atmospheric and room temperature plasma) technique to construct a parental library of *P. anomala* mutants. In the present work, UV mutagenesis yielded 60 single colonies of mutants, which were isolated on selective agar. The survival rate of the mutants was 2.5%. All the isolates were subsequently submitted to a selection procedure in a liquid medium with l-arabinose. During the screening experiment, twenty-eight isolates produced more arabitol than the wild-type strain. Four mutants (M10, M17, M20 and M46) produced arabitol at concentrations of 3.22–3.99 g/L with yields of 0.17–0.24 g/g, amounts that were 41.67–99.5% higher than that produced by the wild-type strain (Fig. 1). In Zhang et al.’s report [21], the best UV mutants of *P. anomala,* U-7 and U-9, showed 7.3 and 8.9% improvements, respectively, in sugar alcohol production from glucose compared to the wild-type strain.

The four mutants with superior arabitol production efficiency were selected for further experiments. Unfortunately, at the selection stage, the wild-type strain produced worse results (Fig. 1) than it did in earlier investigations [23, 24], which could have been a consequence of using a small volume of selective medium without malt extract. Malt extract was not added to this medium to avoid an additional carbon source. However, because the results were comparable with the effectiveness of the mutants, it did not make sense to use higher volumes of the medium for the cultivation of yeasts for selection purposes.

### Genome shuffling

Genome shuffling involves recursive protoplast fusion between multi-parental strains in PEG solution and is aimed at improving the distribution of complex progeny. This method allows for the simultaneous recombination of several genomes at different sites without requiring detailed genomic information [15]. Chemical fusion in a PEG solution has been used for the modification of yeasts, such as *S. cerevisiae* [5], *Pichia* spp. [15, 21], and *Candida* spp. [18, 19]. Protoplast fusion is preceded by the inactivation of parental protoplasts that are divided into two parts and subjected to two different methods: exposure to UV light for 5.5 min or heating at 60 °C for 20 min. These methods are special selection techniques based on the principle of complementary protoplast damage. No colonies grew on the control Petri dishes, indicating that the protoplasts were properly inactivated by UV irradiation and heating, so selection was effective. Genome shuffling, including the protoplast inactivation step, has been used to efficiently increase ethanol tolerance in *S. cerevisiae* [5] and ethanol production from xylose by *P. stipitis* [15].

In the present study, seventeen isolates were obtained on regenerative-selective medium in the first round of genome shuffling of the four parental mutants. The efficiency of the fusants in producing arabitol was confirmed with batch culture. The results for the eight fusants that produced higher concentrations of arabitol than the wild-type strain are shown in Table 1. Only two of the isolates, GSI-1 and GSI-10A, produced concentrations of arabitol that were over 60% higher; these yields were over 50% higher than that of *C. parapsilosis*. They also consumed arabinose faster than the wild-type strain. These two fusants were used for the second round of genome shuffling, which was performed exactly the same as the first round. Fifty colonies were grown on selective medium, and they were all examined microscopically. After inactivation, no colonies of protoplasts grew on control Petri dishes, which also confirms the effectiveness of the selection method. The selection of isolates in liquid medium under the conditions described above showed that fifteen strains were more effective producers of arabitol than the wild-type strain, and only nine of them were better than the two fusants selected from the first round of genome shuffling (Table 2). Eight isolates produced arabitol at concentrations of over 4 g/L (4.10–5.18 g/L) after 2 days of incubation. The best fusants, GSII-16 and GSII-3, produced 5.18 and 4.97 g/L polyol, respectively, giving a yield of 0.33 g/g, which was over 57% higher than that of the wild-type strain. The isolate GSII-16 consumed 77.5% arabinose, which was the best result of all the strains. Similar to the wild-type strain, none of the fusants from the first and second rounds produced other metabolites from l-arabinose. The pH of the cultures of all the selected fusant strains and the wild-type strain ranged from 5 to 5.7. Generally, the fusants from the second round had a higher arabitol production efficiency than those from the first round, but the trends were the same. Similar to the mutant strain cultures, poorer efficiency was caused by the small volume of medium and the semi-optimized conditions used. Batch cultures were maintained monthly (for 1 year) to test the functional stability of the fusants and confirm that the modified microorganisms were stable and produced similar quantities of arabitol from arabinose under the established culture conditions.

**Table 1:** Ability of *C. parapsilosis* fusants obtained in the first round of genome-shuffling to produce arabitol after 2 days of cultivation and the percentage increases in arabitol concentration and yield in relation to those of the wild-type strain

Fusant no.	Residual arabinose concentration (g/L)^a^	Arabitol concentration (g/L)^a^	Increase in arabitol concentration (%)^b^	Arabitol yield (g/g)^a^	Increase in arabitol yield (%)^b^
GSI-1	1.69 ± 0.799	4.15 ± 0.006	66.00	0.23 ± 0.004	53.33
GSI-1A	4.14 ± 0.129	3.21 ± 0.014	28.40	0.20 ± 0.007	33.33
GSI-6A	1.06 ± 0.059	2.79 ± 0.009	11.60	0.15 ± 0.005	0.00
GSI-8A	3.93 ± 0.006	3.18 ± 0.007	27.20	0.20 ± 0.003	33.33
GSI-9A	1.01 ± 0.069	3.35 ± 0.004	34.00	0.18 ± 0.001	20.00
GSI-10A	2.03 ± 0.055	4.09 ± 0.006	63.60	0.23 ± 0.004	53.33
GSI-11A	2.80 ± 0.044	3.53 ± 0.011	41.20	0.21 ± 0.006	40.00
GSI-9B	2.54 ± 0.092	3.32 ± 0.004	32.80	0.19 ± 0.003	26.67
*C. parapsilosis*	3.60 ± 0.148	2.50 ± 0.066	–	0.15 ± 0.042	–

^a^Mean values ± standard deviation from three experiments

^b^Mean values from three experiments

**Table 2:** Ability of *C. parapsilosis* fusants obtained in the second round of genome-shuffling to produce arabitol after 2 days of cultivation and the percentage increases in arabitol concentration and yield in relation to those of the wild-type strain

Fusant no.	Residual arabinose concentration (g/L)^a^	Arabitol concentration (g/L)^a^	Increase in arabitol concentration (%)^b^	Arabitol yield (g/g)^a^	Increase in arabitol yield (%)^b^
GSII-1	6.26 ± 1.455	4.23 ± 0.057	41.47	0.31 ± 0.041	47.62
GSII-3	5.08 ± 2.966	4.97 ± 0.116	66.22	0.33 ± 0.078	57.14
GSII-8	7.09 ± 3.986	4.16 ± 0.132	39.13	0.32 ± 0.102	52.38
GSII-10	5.63 ± 1.503	4.41 ± 0.171	47.49	0.31 ± 0.119	47.62
GSII-12	5.89 ± 2.072	4.24 ± 0.044	41.80	0.30 ± 0.031	42.86
GSII-13	5.73 ± 2.266	4.10 ± 0.057	37.12	0.29 ± 0.040	38.09
GSII-16	4.50 ± 1.049	5.18 ± 0.006	73.24	0.33 ± 0.004	57.14
GSII-24	6.41 ± 3.372	3.92 ± 0.046	31.10	0.29 ± 0.034	38.09
GSII-35	6.23 ± 2.874	4.25 ± 0.051	42.14	0.31 ± 0.037	47.62
GSI-1	5.74 ± 1.871	3.92 ± 0.063	31.10	0.27 ± 0.044	28.57
GSI-10A	5.55 ± 2.238	3.83 ± 0.053	28.09	0.26 ± 0.037	23.81
*C. parapsilosis*	6.02 ± 1.973	2.99 ± 0.006	–	0.21 ± 0.004	–

^a^Mean values ± standard deviation from three experiments

^b^Mean values from three experiments

As previously mentioned, this is the first report of *C. parapsilosis* genome shuffling to improve arabitol production from arabinose. To the best of the authors’ knowledge, there are only two other reports concerning the production of alcohols from sugars by yeast fusants. Zhang et al. [21] obtained *P. anomala* HP fusants that produced arabitol and ribitol in a glucose bioconversion process. Three recombinants of the first round, GS1-1, GS1-2 and GS1-3, exhibited improved productivity, yielding 19.5, 25.6 and 23.9% more total sugar alcohols, respectively, than the wild-type strain. The second-round isolates GS2-1, GS2-2 and GS2-3, obtained by the fusion of GS1-2 and GS1-3, showed increased production levels of total polyalcohols of 46.1, 46.5 and 47.1 g/L, respectively, which were 29.5, 30.6 and 32.2% higher than those of the wild-type strain. The arabitol yields of GS2-1, GS2-2 and GS2-3 were 0.29, 0.31, and 0.32 g/g, respectively, which were 11.5, 19.2 and 23.1% higher, respectively, than those of the original strain *P. anomala* [21]. In a study from Shi et al. [15], *P. stipitis* ATCC 58376 was shuffled to obtain isolates with a better efficiency of ethanol production from xylose. Four positive colonies (TJ2-1 to TJ2-4) were obtained, and the TJ2-3 isolate demonstrated enhanced ethanol production from xylose (2.26% w/v) compared with the wild-type *P. stipitis* (1.63% w/v) and the TJ-1 fusants (1.71–1.9% w/v); this level of production was 38.65% and 18.95–32.16% higher than those of the wild-type *P. stipitis* and the TJ-1 fusants, respectively [15].

### Ploidy of the selected strains

**Fig. 2:**
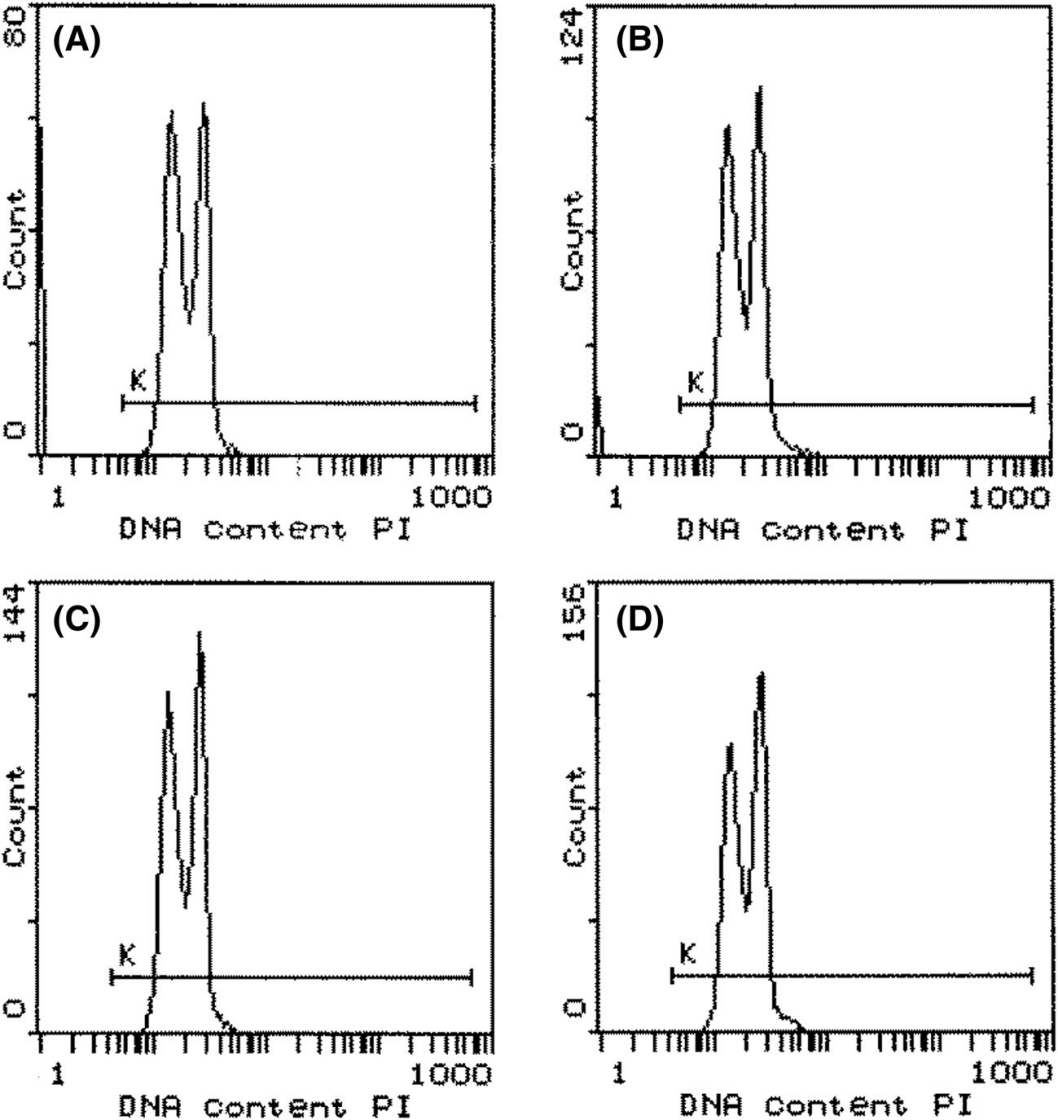
DNA content of wild-type *C. parapsilosis* (**A**) and the fusants GSI-1 (**B**), GSII-3 (**C**), and GSII-16 (**D**) determined by flow cytometry. Peaks in the histograms show pre-replication and post-replication cells

The DNA content of the yeast cells is measured by the intensity of red fluorescence they emit. The DNA content of the wild-type strain and the selected fusants after the first and second rounds of genome shuffling was determined to compare the ploidy of the new isolates. No significant differences in DNA concentrations and ploidy between the fusants and the control strain were observed (Fig. 2). These results suggest that the physiological differences between the studied strains could be due to the gene exchange caused by homologous recombination during genome shuffling and not the presence of additional chromosomes. Hou [7] and Wang and Hou [10] made the same observations for genome-shuffled *S. cerevisiae*, and Cao et al. [20] made the same observations for *H. anomala* using the same FCM method. Additionally, Wei et al. [18] and Cao et al. [19], who conducted genome shuffling experiments on *C. krusei* and *C. versatilis*, respectively, did not observe an increase in DNA content using a diphenylamine assay, which indicated that the complete addition of the chromosomes of the wild-type strain did not occur.

### Arabitol production under optimal conditions

**Fig. 3:**
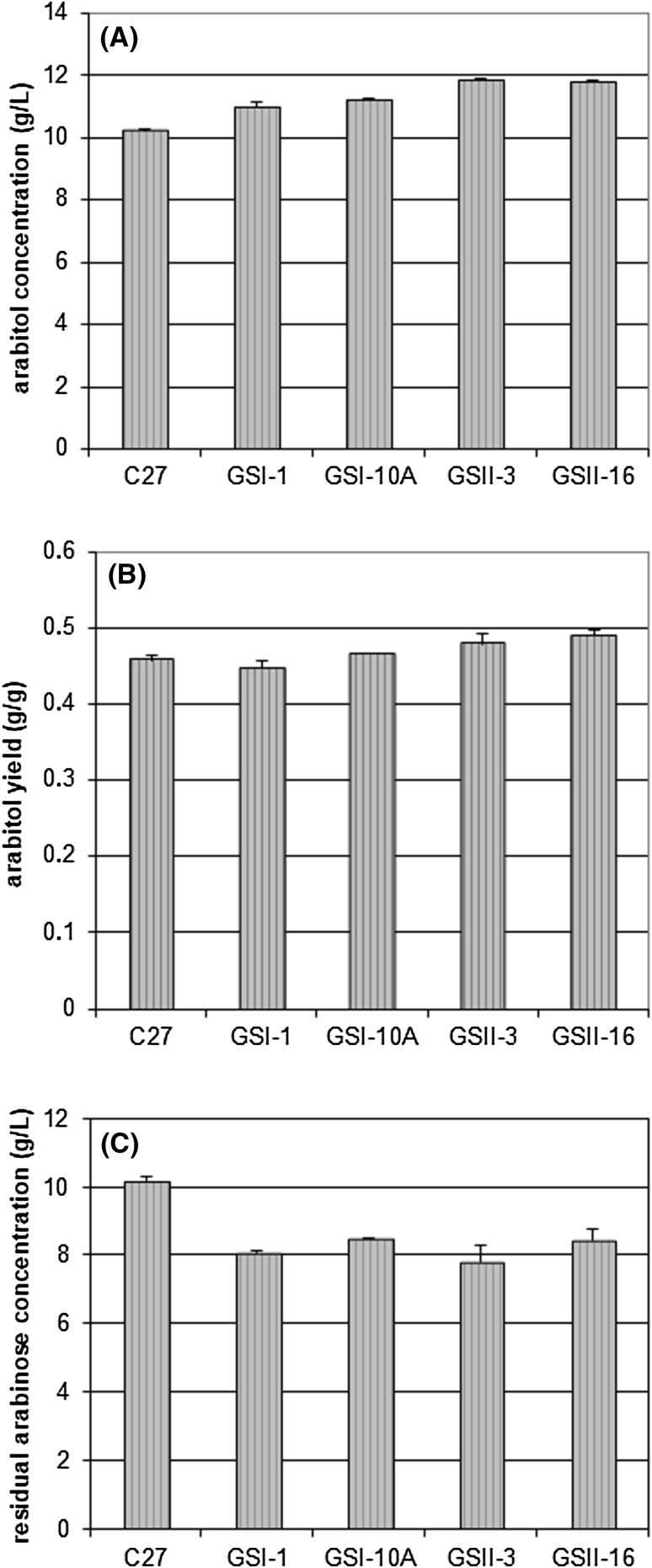
Comparison of arabitol production (**A**), arabitol yield (**B**) and arabinose utilization (**C**) between the fusants of the first and second rounds of genome shuffling and the wild-type strain *C. parapsilosis* DSM 70125 under optimal conditions. The mean values are from three experiments

The most efficient fusants, GSII3 and GSII16, were cultivated in the optimal medium under conditions described by Kordowska-Wiater et al. [24]. GSII3 and GSII16 produced arabitol at concentrations of 11.83 and 11.75 g/L, respectively, and were thus approximately 15–16% more efficient than the wild-type strain [Fig. 3(A)]. The yields obtained (0.479 and 0.489 g/g for GSII3 and GSII16, respectively) were also higher than those produced by the wild-type strain [Fig. 3(B)]. The best first-round fusants showed medium efficiency. All fusants metabolized arabinose faster than *C. parapsilosis* [Fig. 3(C)]. The biomass concentrations (6.17–6.87 g/L) and biomass yield (0.25–0.29 g/g) after the specified incubation time were very similar for the wild-type and modified strains. The pH fell to 3.84–3.92. These data confirm earlier observations and demonstrate that the modified yeast cells can be successfully applied for arabitol production from pure arabinose. It is important to note, however, that the efficiency of the process was strictly dependent on the volume of the culture and, thus, the oxygen availability. In the small volumes of medium (20 mL) used at the selection step, the wild-type yeasts produced approximately three times less arabitol, and the fusants produced approximately 2–2.5 times less arabitol than they did in a larger (100 mL) volume of optimal medium.

In conclusion, genome shuffling is an interesting and effective method for improving yeast for biotechnological purposes. The new isolates obtained were proven to produce arabitol from l-arabinose at higher concentrations and yields than *C. parapsilosis* DSM 70125. The fact that the selected isolates and the wild-type strain had a similar DNA content suggests that the metabolic differences could have been an effect of gene exchange during genome shuffling rather than the presence of additional chromosomes. In the selection step, the best fusants produced arabitol at low concentrations (approximately 4–5 g/L), which, nevertheless, were over 60% higher than those produced by the wild-type strain. By contrast, under optimal culture conditions, the fusants secreted over 11 g/L arabitol, a concentration that was only 15–16% higher than that produced by the wild-type strain. These results suggest that further optimization studies are necessary and that every fusant may require special, isolate-specific conditions to become an effective producer of arabitol.
